# Consumer Acceptability and Sensory Profile of Three New Celery (*Apium graveolens*) Hybrids and Their Parental Genotypes

**DOI:** 10.3390/ijms222413561

**Published:** 2021-12-17

**Authors:** Lucy Turner, Carol Wagstaff, Frances Gawthrop, Stella Lignou

**Affiliations:** 1 Department of Food and Nutritional Sciences, Harry Nursten Building, University of Reading, Whiteknights, Reading RG6 6DZ, UK; L.Turner@pgr.reading.ac.uk (L.T.); c.wagstaff@reading.ac.uk (C.W.); 2 A.L. Tozer Ltd., Pyports, Downside Bridge Road, Cobham KT11 3EH, UK; frances.gawthrop@tozerseeds.com

**Keywords:** celery, volatiles, flavour, sensory perception, consumer liking, postharvest, terpene, phthalides

## Abstract

Celery is a stalky green vegetable that is grown and consumed globally and used in many cuisines for its distinctive taste and flavour. Previous investigations identified the aroma composition of celery and profiled its sensory characteristics using a trained panel; however, evaluation of the sensory characteristics of celery combined with a consumer panel, where consumer preferences and acceptability are determined, is novel. In this study, three parental genotypes (12, 22 and 25) and three new hybrids (12x22, 22x12 and 25x12) were presented to a trained sensory panel (*n* = 12) for profiling and a consumer panel (*n* = 118), where liking and preference were assessed. Celery samples were analysed by SPME GC–MS and significant differences in aroma composition between all samples were identified, causing significant differences in the sensory profile. Furthermore, significant differences in attributes assessed for liking (appearance, aroma, texture and overall) were identified. Consumer segmentation identified three groups of consumers exhibiting differences in the hedonic reaction to the samples. Sweet and bitter taste along with overall flavour were identified as drivers of liking. Hybrid 25x12 was found to be the hybrid that exhibited high intensities for most of the attributes assessed.

## 1. Introduction

Celery is an aromatic vegetable that is grown and consumed globally in a range of salads, with condiments; in cooking, where it can be boiled, fried, roasted as well as forming the base of many soups, stocks, and sauces [1,2,3]. Within cuisines, celery is known to form part of the holy trinity or soffritto [3], starring alongside carrots and onions or onions and bell peppers depending on the cuisine. Celery owes its culinary diversity to the distinct aroma and flavour profile, possessing a range of compound groups including terpenes (monoterpenes and sesquiterpenes), alcohols, aldehydes and phthalides contributing to the overall flavour quality of celery [3,4,5,6,7,8]. The phthalide compounds have been established as the characteristic odorants of celery, with odour descriptors such as ‘celery’, ‘cooked celery’ and ‘herbal’. Without the presence of these compounds, celery aroma would not be so distinctive [7,9].

Being such a commonly grown and consumed vegetable, research investigating the perception of celery flavour is surprisingly sparse, with only a few sources examining the sensory properties of celery [9,10,11,12,13]. Furthermore, there has been no research conducted that explores the sensory characteristics of celery combined with consumers’ perceptions and preferences. Previous research has identified that external characteristics such as product appearance are primary influencers of initial consumer purchase, whilst internal characteristics that follow consumption (aroma, taste, flavour, texture) influence acceptability and repurchase [14,15,16]. Without completing sensory and consumer evaluation, the acceptability of celery and the sensory characteristics that consumers find desirable within celery remain unknown and crop breeding programmes are missing key information that should direct their selection processes. 

The authors have previously carried out several experiments, where they identified the aroma profile of various celery genotypes and investigated how factors such as genotype, maturity, geographical location, climate, and agronomy influence the aroma profile and the sensory characteristics using a trained panel [9,12,13]. Combining data from instrumental and sensory analysis with multi-site and multi-year investigations that use the same eight genotypes has led to the discovery of three genotypes that consistently performed regardless of influencing environmental or developmental factors; genotypes 12, 22 and 25. Genotype 12 was consistently high in the abundance of volatile compounds with a high percentage of phthalides comprising the aroma profile of celery with a strong, typical celery odour. The trained panel strongly associated this genotype with a grass odour and herbal flavour, including fennel, parsley, and coriander [9,12,13]. On the other hand, genotype 25 exhibited low abundance of phthalides and a high abundance of aldehydes, with the trained panel describing this genotype as having a cucumber flavour. Genotype 22 had similar aroma profile to genotype 12 but with lower abundance and was scored lower by the trained panel for aroma and flavour attributes such as fresh parsley, coriander, and fennel. In terms of mouthfeel, genotype 22 was consistently scored high for a moist and crunchy petiole and low for stringy mouthfeel, opposing genotype 12. Genotype 12 was ribbed, stringy and bitter, genotypes 22 and 25 remained crunchy, moist with minimal stringiness [12,13].

Providing celery growers and breeders with the information gathered from this investigation will aid in the development of new celery hybrids that have been tailor-made according to consumer preference. The aim of this study was to evaluate the sensory characteristics of celery parental genotypes (12, 22 and 25) and their hybrids (12x22, 25x12 and 22x12) using a trained sensory panel and to assess the aroma profile of the same samples using solid-phase microextraction gas chromatography–mass spectrometry (SPME GC–MS) to identify differences and similarities within the aroma profile. Consumer evaluation was also conducted to understand the acceptability, liking and preference of these genotypes and hybrids and to associate sensory and biochemical composition with these desirable characteristics.

## 2. Results and Discussion

### 2.1. Volatile Composition of Celery Samples

In total, 100 compounds were identified in the headspace of the six celery samples (Table 1) including 28 monoterpenes, 16 sesquiterpenes, 12 alcohols (five of which are classified as monoterpenoid alcohols), nine aldehydes and five phthalides. Quantitative differences were observed between the genotypes used in this study and one-way ANOVA revealed significant differences in the relative abundance of aroma compounds between the genotypes in most compounds. Compounds such as (*E*)-2-penten-1-ol, (*Z*)-3-hexenol, lavandulyl acetate, δ-3-carene, β-thujone, *p*-1,3,8-menthatriene, fenchol and β-eudesmol expressed no significant difference between genotypes accompanied by several alkanes and unknown compounds.

A large proportion of the aroma profile was comprised of monoterpenes and sesquiterpenes with limonene, β-pinene, myrcene, γ-terpinene and β-caryophyllene exhibiting the highest relative abundance within their compound groups. These compounds are commonly present in celery and have been reported to contribute to odour notes such as woody, herbal, green, waxy, and earthy [3,9]. Monoterpenes have been shown to have the highest proportion of the aroma composition in various studies [3,5,6]. Genotype 12 exhibited the highest abundance of monoterpenes, sesquiterpenes and phthalides, followed by hybrids 22x12 and 12x22, while genotype 25 and hybrid 25x12 had a much lower abundance of these compounds. However, as reported by the authors, these terpenes are not the characteristic compounds in celery [4].

Sesquiterpenes, whilst at a lower relative abundance to monoterpenes are more typical to the mature celery aroma. Previously reported by the authors [9], during maturation, the celery aroma developed significantly, starting as a fresh, citrus, green aroma due to the high proportion of monoterpenes and lack of sesquiterpene and phthalide compounds. As the celery matured, the abundance of sesquiterpenes and phthalides became much more apparent and thus, a change in the perceived aroma was identified [9]. β-Caryophyllene and β-selinene (Table 1) exhibited the highest relative abundance within all genotypes, and this was most obviously observed in genotype 12 and hybrid 22x12. Ehiabhi et al. [17] reported β-caryophyllene and β-selinene to be major constituents of Nigerian grown celery and Lund, Wagner, and Bryan [18] identified β-selinene to impart a strong celery aroma. Although less abundant in other genotypes, genotype 12 had a high abundance of kessane. Kessane was identified by Philippe, Suvarnalatha, Sankar and Suresh [19] in the essential oil of Indian-grown celery seed, comprising between 2.2 and 7.6% of the volatile profile.

Phthalides have been shown to contribute to strong celery-like odours in addition to being the most odour-active compounds within celery crop. Upon completing aroma extraction dilution analysis (AEDA), Kurobayashi [20] detected phthalide compounds including 3-n-butylphthalide and sedanenolide, also identified within this study, to contribute most to celery odour. This was further confirmed by Lund, Wagner and Bryan [18], whereby sedanenolide, 3-n-butylphthalide and hexahydro-3-n-butylphthalide imparted strong celery odour characteristics. Genotype 12 displayed the highest abundance of phthalide compounds (Table 1) including sedanenolide and 3-n-butylphthalide followed by hybrids 12x22 and 22x12 that also displayed a high abundance of phthalides within their aroma profile. As these compounds consist of strong celery odour notes [8], we can assume these celeries consist of a typical celery flavour.

The maternal inheritance of compounds from parent to hybrid was observed most clearly between genotype 25 and hybrid 25x12, whereby similarities between the presence and absence of compounds within the aroma profile as well as the abundance of compounds was apparent (Table 1). Monoterpene, sesquiterpene and phthalide abundances for these celery samples were the lowest out of the six samples and for example camphor and p-mentha-2,8-diene were both not identified in genotype 25 and 25x12. Furthermore, apart from 3-propylidene phthalide, the relative abundances of phthalide compounds were not significantly different between 25 and 25x12. The influence of the female counterpart of the crop is clear, with 25x12 inheriting more similarities from the female parent, 25 than male parent 12. This is less clearly observed when both parents, 12 and 22, were used in the hybrids 12x22 and 22x12. The relationship of these genotypes is unknown but if there is a close relation, genetically, then this would explain the fewer significant differences observed between these hybrids (Table 1). m-Tolualdehyde was only identified in genotype 22 and hybrid 22x12 and other aldehydes such as (E, E)-2,4-octadienal and hexanal were either only expressed in 12, 12x22 and 22x12 or were expressed in high abundance in these samples. The chemical inheritance of monoterpenes and sesquiterpene compounds appeared to be less clear; however, β-selinene and β-caryophyllene were expressed in a high relative abundance in genotype 12 and hybrid 22x12, displaying a stronger influence from the male parent, 12. Genotype 12 also displayed a high influence over the phthalide content for the hybrids 12x22 and 22x12, where both expressed a higher relative abundance for phthalide compounds than genotype 22.

**Table 1:** Relative abundance of aroma compounds identified in the headspace of fresh celery samples.

Code	Compound Name	LRI ^a^	ID ^b^	Relative Abundance (AU) ^c^						*p*-Value
				12	22	25	25x12	12x22	22x12	
	**Alcohols**									
A1	(*E*)-2-penten-1-ol	758	A	nd	0.53 ± 0.74	0.43 ± 0.05	nd	nd	0.83 ± 0.09	ns
A2	pentanol	762	A	nd ^b^	nd ^b^	nd ^b^	0.48 ± 0.14 ^ab^	0.68 ± 0.33 ^a^	0.15 ± 0.21 ^ab^	**
A3	(*Z*)-3-hexenol	849	B [21]	4.1 ± 2.5 ^a^	4.1 ± 1.7	nd	2.0 ± 0.47	4.3 ± 1.1	1.2 ± 0.18	ns
A4	(*E*)-3-hexenol	852	A	6.2 ± 2.9 ^a^	3.5 ± 1.8 ^ab^	1.3 ± 0.26 ^b^	nd ^b^	3.7 ± 0.53 ^ab^	0.69 ± 0.49 ^b^	*
A5	hexanol	862	A	nd ^b^	nd ^b^	0.53 ± 0.03 ^b^	0.65 ± 0.04 ^b^	3.0 ± 0.98 ^a^	3.6 ± 1.1 ^a^	***
A6	octanol	1072	A	4.9 ± 0.70 ^ab^	5.3 ± 0.61 ^a^	1.3 ± 0.13 ^cd^	nd ^d^	2.9 ± 1.2 ^bc^	3.8 ± 0.36 ^ab^	***
A7	(*Z*)-3-nonenol	1153	B [22]	5.6 ± 2.9	6.1 ± 2.6	1.8 ± 0.81	1.3 ± 0.16	6.9 ± 1.7	5.9 ± 0.98	*
	**Aldehydes**									
AL1	hexanal	800	A	9.23 ± 0.33 ^ab^	0.43 ± 0.06 ^b^	0.15 ± 0.12 ^b^	0.30 ± 0.05 ^b^	0.46 ± 0.31 ^b^	91 ± 18 ^a^	***
AL2	benzaldehyde	964	A	nd ^b^	nd ^b^	nd ^b^	nd ^b^	0.24 ± 0.04 ^a^	nd ^b^	***
AL3	octanal	1008	A	7.6 ± 1.4 ^ab^	9.5 ± 2.4 ^a^	3.6 ± 0.62 ^bc^	2.4 ± 0.58 ^c^	5.3 ± 1.3 ^abc^	9.4 ± 1.1 ^a^	**
AL4	benzeneacetaldehyde	1058	A	6.4 ± 1.3 ^a^	6.5 ± 2.4 ^a^	1.9 ± 0.25 ^bc^	0.96 ± 0.43 ^c^	3.7 ± 1.6 ^abc^	5.2 ± 0.60 ^ab^	**
AL5	m-tolualdehyde	1083	B [23]	nd ^b^	19 ± 2.4 ^a^	nd ^b^	nd ^b^	nd ^b^	16 ± 1.2 ^a^	***
AL6	(*E*,*E*)-2,4-octadienal	1116	A	2.0 ± 1.1 ^b^	nd ^b^	nd ^b^	nd ^b^	1.6 ± 0.57 ^b^	4.2 ± 0.72 ^a^	***
AL7	(*E*,*E*)-2,6-nonadienal	1155	A	2.3 ± 1.6	nd	nd	0.39 ± 0.55	nd	nd	*
AL8	(*E*)-2-nonenal	1171	A	3.2 ± 0.44 ^a^	2.7 ± 0.46 ^a^	0.69 ± 0.09 ^b^	0.89 ± 0.14 ^b^	0.69 ± 0.97 ^b^	1.8 ± 0.07 ^ab^	***
AL9	undecanal	1306		nd ^c^	nd ^c^	0.93 ± 0.28 ^bc^	1.4 ± 0.35 ^bc^	1.6 ± 0.44 ^b^	3.8 ± 0.79 ^a^	***
	**Esters**									
E1	allyl hexanoate	1080	A	3.9 ± 0.62 ^ab^	nd ^c^	2.0 ± 0.43 ^bc^	1.2 ± 0.92 ^bc^	3.1 ± 0.96 ^ab^	6.0 ± 1.5 ^a^	***
E2	(*E*,*Z*)-3,6 nonadienol acetate	1174	B [24]	4.4 ± 0.45 ^a^	2.2 ± 0.49 ^bc^	1.0 ± 0.12 ^c^	1.5 ± 0.15 ^c^	2.2 ± 0.41 ^bc^	3.3 ± 0.48 ^ab^	***
E3	(*Z*)-3-hexenyl butanoate	1185	A	2.5 ± 0.23 ^b^	2.6 ± 0.10 ^b^	nd ^d^	nd ^d^	1.3 ± 0.45 ^c^	4.5 ± 0.54 ^a^	***
E4	lavandulyl acetate	1285	B [25]	0.34 ± 0.48	0.72 ± 0.20	0.15 ± 0.22	0.64 ± 0.14	0.15 ± 0.22	1.1 ± 0.79	ns
	**Ketones**									
K1	acetophenone	1077	A	8.4 ± 1.1 ^a^	nd ^b^	1.8 ± 0.26 ^b^	0.68 ± 0.35 ^b^	8.2 ± 0.86 ^a^	14 ± 1.5 ^a^	***
K2	(*Z*)-jasmone	1405	A	2.3 ± 0.38 ^a^	0.24 ± 0.33 ^c^	0.48 ± 0.04 ^bc^	0.10 ± 0.15 ^c^	nd ^c^	0.99 ± 0.05 ^b^	***
	**Alkanes**									
AK1	nonane	897	A	17 ± 2.8 ^b^	46 ± 1.9 ^a^	8.4 ± 1.5 ^b^	19 ± 1.1 ^b^	21 ± 1.6 ^b^	52 ± 11 ^a^	***
AK2	decane	998	A	nd ^c^	10 ± 3.5 ^ab^	4.9 ± 0.93 ^bc^	5.0 ± 0.93 ^bc^	6.3 ± 3.2 ^bc^	14 ± 1.3 ^a^	***
AK3	undecane	1097	A	27 ± 9.6	23 ± 11.2	10 ± 2.1	9.3 ± 1.9	12 ± 4.1	22 ± 5.1	ns
AK4	dodecane	1197	A	14 ± 9.6	6.3 ± 3.6	1.5 ± 0.65	2.9 ± 0.85	4.5 ± 1.2	6.8 ± 0.60	ns
AK5	tridecane	1297	A	18 ± 1.2	4.0 ± 3.8	1.1 ± 0.20	1.1 ± 0.92	1.7 ± 1.3	1.9 ± 1.2	ns
AK6	tetradecane	1397	A	40 ± 1.5	9.5 ± 7.9	3.2 ± 1.8	2.7 ± 2.0	4.6 ± 3.5	5.5 ± 2.8	ns
AK7	pentadecane	1498	A	35 ± 9.1	9.3 ± 6.1	3.3 ± 0.84	3.3 ± 1.9	6.0 ± 3.9	3.2 ± 2.3	ns
AK8	hexadecane	1599	A	17 ± 11	4.6 ± 2.2	1.7 ± 0.71	1.8 ± 0.84	3.4 ± 1.8	4.0 ± 1.3	ns
AK9	heptadecane	1699	A	8.2 ± 2.6 ^a^	2.3 ± 0.49 ^b^	0.99 ± 0.08 ^b^	1.0 ± 0.20 ^b^	2.2 ± 1.1 ^b^	2.8 ± 0.13 ^b^	***
AK10	octadecane	1800	A	nd	0.76 ± 0.20	0.13 ± 0.19	0.25 ± 0.19	0.32 ± 0.45	0.75 ± 0.17	*
	**Monoterpenes**									
M1	α-thujene	932	B [26]	10 ± 1.8 ^a^	4.8 ± 0.42 ^b^	2.7 ± 0.39 ^b^	3.7 ± 0.49 ^b^	4.2 ± 0.49 ^b^	5.0 ± 0.45 ^b^	***
M2	α-pinene	941	A	22 ± 2.9 ^a^	24 ± 2.1 ^a^	6.2 ± 0.97 ^b^	8.5 ± 0.80 ^b^	19 ± 1.8 ^a^	20 ± 2.8 ^a^	***
M3	camphene	958	A	5.6 ± 0.59 ^a^	6.0 ± 1.3 ^a^	2.0 ± 0.13 ^b^	2.5 ± 0.25 ^b^	4.3 ± 0.46 ^ab^	5.4 ± 0.81 ^a^	***
M4	sabinene	980	A	34 ± 5.5 ^a^	18 ± 5.9 ^b^	5.8 ± 1.1 ^b^	8.7 ± 1.3 ^b^	12 ± 1.1 ^b^	19 ± 6.8	**
M5	β-pinene	987	A	110 ± 15 ^ab^	122 ± 23 ^ab^	70 ± 12 ^b^	86 ± 12 ^b^	120 ± 8.2 ^ab^	145 ± 23 ^a^	**
M6	myrcene	990	A	799 ± 67 ^a^	100 ± 9.0 ^bcd^	42 ± 4.4 ^d^	59 ± 7.7 ^cd^	149 ± 24 ^bc^	173 ± 25 ^b^	***
M7	*p*-mentha-2,8-diene	1005	B [27]	2.5 ± 1.1	5.2 ± 0.89	nd	nd	3.3 ± 1.1	4.3 ± 0.64	*
M8	α-phellandrene	1013	A	19 ± 2.6 ^a^	14 ± 2.6 ^ab^	6.3 ± 0.87 ^c^	5.5 ± 1.1 ^c^	9.6 ± 2.1 ^bc^	17 ± 0.80 ^a^	***
M9	δ-3-carene	1019	A	1.2 ± 1.6	nd	nd	0.82 ± 0.19	nd	nd	ns
M10	α-terpinene	1024	A	30 ± 5.6 ^a^	14 ± 1.9 ^b^	8.0 ± 0.89 ^b^	11 ± 3.0 ^b^	8.1 ± 2.7 ^b^	14 ± 2.4 ^b^	***
M11	*o*-cymene	1030	A	469 ± 11 ^a^	190 ± 22 ^de^	128 ± 20 ^e^	213 ± 0.16 ^cd^	299 ± 37 ^b^	267 ± 14 ^bc^	***
M12	limonene	1037	A	6524 ± 207 ^a^	3259 ± 236 ^b^	1188 ± 89 ^d^	1285 ± 84 ^d^	2371 ± 246 ^c^	3638 ± 441 ^b^	***
M13	β-(*E*)-ocimene	1048	B [28]	54 ± 6.2 ^a^	63 ± 2.3 ^a^	13 ± 0.89 ^c^	5.1 ± 0.95 ^c^	34 ± 8.6 ^b^	45 ± 7.2 ^ab^	***
M14	γ-terpinene	1065	A	1455 ± 112 ^a^	732 ± 127 ^b^	329 ± 39 ^c^	539 ± 96 ^bc^	389 ± 89 ^bc^	689 ± 179 ^bc^	***
M15	*p*-cymenene	1095	A	nd ^b^	19 ± 2.6 ^a^	nd ^b^	nd ^b^	nd ^b^	7.0 ± 9.9 ^ab^	**
M16	terpinolene	1096	A	38 ± 4.6 ^a^	nd ^c^	7.0 ± 0.48 ^bc^	6.5 ± 1.0 ^bc^	14 ± 3.9 ^b^	11 ± 7.6 ^bc^	***
M17	β-thujone	1119	A	1.9 ± 1.3	0.58 ± 0.82	0.45 ± 0.32	0.13 ± 0.18	nd	nd	ns
M18	allo-ocimene	1130	B [29]	150 ± 16 ^ab^	177 ± 13 ^a^	30 ± 3.2 ^c^	9.2 ± 0.74 ^c^	106 ± 20 ^b^	144 ± 17 ^ab^	***
M19	*p*-1,3,8 menthatriene	1134	B [30]	6.2 ± 8.7	11 ± 7.7	2.4 ± 1.7	1.2 ± 0.05	13 ± 2.0	8.7 ± 6.1	ns
M20	trans-allo-ocimene	1144	B [31]	81 ± 5.9 ^a^	79 ± 8.6 ^a^	20 ± 2.3 ^bc^	12 ± 2.9 ^c^	42 ± 11 ^b^	78 ± 11 ^a^	***
M21	camphor	1157	A	nd ^c^	2.2 ± 0.16 ^b^	nd ^c^	nd ^c^	1.9 ± 0.39 ^b^	3.2 ± 0.28 ^a^	***
M22	pentylcyclohexa-1,3-diene	1161	B [32]	3.3 ± 0.64 ^b^	5.4 ± 1.2 ^b^	16 ± 1.1 ^ab^	17 ± 2.0 ^ab^	56 ± 13 ^a^	25 ± 7.1 ^ab^	*
M23	*trans*-dihydrocarvone	1206	A	4.1 ± 0.95 ^a^	1.9 ± 0.41 ^b^	1.3 ± 0.86 ^b^	0.91 ± 0.19 ^b^	1.9 ± 0.34 ^b^	2.7 ± 0.32 ^ab^	**
M24	safranal	1215	A	11 ± 2.6 ^a^	4.6 ± 0.69 ^bc^	1.5 ± 0.63 ^c^	2.5 ± 0.68 ^c^	2.7 ± 0.98 ^c^	7.9 ± 0.44 ^ab^	***
M25	β-cyclocitral	1235	A	3.6 ± 0.79 ^a^	1.9 ± 0.50 ^ab^	0.73 ± 0.19 ^b^	1.0 ± 0.29 ^b^	0.81 ± 0.61 ^b^	3.5 ± 0.35 ^a^	***
M26	L-carvone	1251	A	2.5 ± 0.86 ^ab^	2.1 ± 0.57 ^ab^	nd ^c^	0.89 ± 0.18 ^bc^	1.5 ± 0.39 abc	2.9 ± 0.64 ^a^	***
M27	D-carvone	1259	A	3.5 ± 0.31	2.9 ± 1.2	1.5 ± 0.51	1.4 ± 0.23	1.7 ± 0.39	3.4 ± 0.77	*
M28	carvacrol	1318	A	nd ^b^	nd ^b^	0.12 ± 0.17 ^b^	0.42 ± 0.09 ^b^	0.51 ± 0.39 ^ab^	1.1 ± 0.15 ^a^	**
	**Monoterpenoid Alcohols**									
MA1	(+)-*cis*-*p*-mentha-2,8-dien-1-ol	1124	A	5.0 ± 1.1 ^a^	5.5 ± 0.35 ^a^	0.95 ± 0.17 ^b^	0.15 ± 0.21 ^b^	4.7 ± 0.97 ^a^	4.0 ± 0.15 ^a^	***
MA2	fenchol	1127	A	0.55 ± 0.76	nd	nd	0.14 ± 0.19	nd	0.87 ± 0.64	ns
MA3	*trans*-carveol	1225	B [33]	9.8 ± 4.5 ^a^	1.9 ± 0.18 ^c^	0.99 ± 0.10 ^d^	1.4 ± 0.10 ^cd^	1.7 ± 0.13 ^c^	3.0 ± 0.26 ^b^	***
MA4	*cis*-carveol	1238	A	3.3 ± 0.10 ^a^	2.3 ± 0.18 ^a^	0.63 ± 0.48 ^b^	0.63 ± 0.18 ^b^	0.45 ± 0.63 ^b^	2.6 ± 0.16 ^a^	***
MA5	(*Z*)-8-hydroxy linalool	1346	B [34]	2.7 ± 0.43 ^a^	0.76 ± 0.08 ^c^	0.27 ± 0.19 ^c^	0.59 ± 0.14 ^c^	0.50 ± 0.37 ^c^	1.7 ± 0.12 ^b^	***
	**Sesquiterpenes**									
S1	α-ylangene	1387	B [35]	3.1 ± 1.1 ^a^	3.0 ± 0.65 ^a^	1.7 ± 0.16 ^ab^	0.69 ± 0.09 ^b^	1.1 ± 0.39 ^b^	1.8 ± 0.17 ^ab^	**
S2	α-copaene	1392	A	nd ^e^	9.2 ± 0.11 ^a^	6.2 ± 0.18 ^b^	2.0 ± 0.18 ^d^	1.8 ± 0.30 ^d^	4.5 ± 0.43 ^c^	***
S3	(*E*)-β-caryophyllene	1427	B [31]	2.2 ± 0.42 ^a^	0.25 ± 0.35 ^b^	0.49 ± 0.05 ^b^	0.33 ± 0.07 ^b^	nd ^b^	0.87 ± 0.68 ^b^	**
S4	β-caryophyllene	1442	A	217 ± 9.8 ^a^	71 ± 1.3 ^c^	60 ± 1.2 ^cd^	46 ± 4.5 ^d^	44 ± 8.4 ^d^	97 ± 11 ^b^	***
S5	(+)-aromadend rene	1461	A	2.2 ± 0.10 ^ab^	1.2 ± 0.38 ^cd^	2.7 ± 0.42 ^a^	0.21 ± 0.30 ^d^	0.98 ± 0.32 ^cd^	1.5 ± 0.14 ^bc^	***
S6	curcumene	1470	B [36]	3.3 ± 0.15 ^a^	nd ^b^	0.78 ± 0.11 ^b^	0.72 ± 0.13 ^b^	nd ^b^	0.59 ± 0.83 ^b^	***
S7	α-humulene	1477	A	19 ± 1.2 ^a^	12 ± 0.69 ^b^	4.5 ± 0.10 ^c^	6.3 ± 0.66 ^c^	6.1 ± 1.3 ^c^	11 ± 0.89 ^b^	***
S8	γ-himachalene	1493	B [33]	2.8 ± 0.33 ^a^	2.1 ± 0.16 ^ab^	1.1 ± 0.05 ^c^	0.92 ± 0.14 ^c^	1.3 ± 0.35 ^bc^	2.3 ± 0.19 ^a^	***
S9	β-selinene	1511	B [33]	192 ± 14 ^a^	31 ± 0.93 ^c^	24 ± 0.82 ^c^	24 ± 1.9 ^c^	29 ± 4.7 ^c^	59 ± 4.9 ^b^	***
S10	valencene	1515	A	261 ± 31 ^a^	3.5 ± 1.5 ^b^	3.6 ± 0.16 ^b^	1.6 ± 0.16 ^b^	34 ± 4.4 ^b^	33 ± 2.4 ^b^	***
S11	α-selinene	1519	B [32]	22 ± 1.3 ^a^	5.4 ± 0.16 ^bc^	3.7 ± 0.19 ^c^	3.2 ± 0.27 ^c^	3.8 ± 0.64 ^c^	7.4 ± 0.71 ^b^	***
S12	(*E*)*-*nerolidol	1540	B [37]	nd ^d^	2.3 ± 0.19 ^a^	1.7 ± 0.05 ^b^	0.91 ± 0.21 ^c^	0.21 ± 0.29 ^d^	1.2 ± 0.11 ^bc^	***
S13	kessane	1555	B [32]	200 ± 39 ^a^	2.3 ± 0.30 ^b^	0.51 ± 0.04 ^b^	0.51 ± 0.09 ^b^	26 ± 3.1 ^b^	27 ± 1.9 ^b^	***
S14	liguloxide^$^	1561	B [38]	5.2 ± 0.89 ^a^	nd ^b^	nd ^b^	nd ^b^	0.67 ± 0.11 ^b^	0.66 ± 0.47 ^b^	***
S15	rosifoliol	1588	B [39]	nd ^c^	0.45 ± 0.32 ^abc^	0.16 ± 0.23 ^bc^	0.70 ± 0.09 ^ab^	0.41 ± 0.29 ^abc^	0.99 ± 0.04 ^a^	**
S16	β-eudesmol	1633	B [40	nd	nd	nd	0.29 ± 0.19	0.65 ± 0.92	nd	ns
	**Oxides**									
O1	caryophyllene oxide	1608	A	2.0 ± 0.26 ^a^	0.30 ± 0.23 ^d^	0.39 ± 0.05 ^d^	0.59 ± 0.08 ^cd^	1.2 ± 0.02 ^bc^	1.7 ± 0.23 ^ab^	***
	**Phthalides**									
P1	3-propylidene phthalide	1603	A	7.7 ± 0.91 ^a^	0.87 ± 0.37 ^b^	0.54 ± 0.03 ^b^	nd ^b^	0.46 ± 0.33 ^b^	nd ^b^	***
P2	3-n-butylphthalide	1675	B [9,12,13]	18 ± 7.8 ^a^	8.7 ± 2.9 ^ab^	3.8 ± 1.3 ^b^	3.4 ± 0.70 ^b^	13 ± 1.4 ^ab^	13 ± 1.7 ^ab^	*
P3	sedanenolide	1747	B [9,12,13]	58 ± 4.0 ^a^	16 ± 2.9 ^c^	5.2 ± 0.50 ^d^	4.5 ± 0.35 ^d^	25 ± 3.4 ^b^	21 ± 2.2 ^bc^	***
P4	*trans*-neocnidilide	1754	B [32]	2.7 ± 0.24 ^a^	2.8 ± 0.33 ^a^	1.3 ± 0.12 ^b^	1.8 ± 0.08 ^b^	2.7 ± 0.05 ^a^	2.9 ± 0.19 ^a^	***
P5	(*Z*)*-*ligustilide	1763	B [9,12,13]	4.0 ± 0.49 ^a^	0.41 ± 0.08 ^b^	0.21 ± 0.08 ^b^	0.24 ± 0.04 ^b^	1.0 ± 0.79 ^b^	0.77 ± 0.10 ^b^	***
	**Unknowns**									
U1	unknown 1	840		2.6 ± 0.79	nd	3.1 ± 0.71	2.0 ± 0.23	nd	4.5 ± 3.5	ns
U2	unknown 2	1076		nd ^b^	19 ± 5.5 ^a^	nd ^b^	nd ^b^	nd ^b^	nd ^b^	***
U3	unknown 3	1084		15 ± 2.0 ^a^	nd ^b^	nd ^b^	2.7 ± 0.54 ^b^	11 ± 3.3 ^a^	nd ^b^	***
U4	unknown 4	1141		2.2 ± 0.38 ^a^	1.4 ± 0.98 ^ab^	nd ^b^	0.30 ± 0.25 ^ab^	1.6 ± 0.35 ^ab^	1.4 ± 0.98 ^ab^	*
U5	unknown 5	1189		1.2 ± 1.7	0.62 ± 0.88	1.2 ± 1.7	0.15 ± 0.21	0.35 ± 0.49	nd	ns
U6	unknown 6	1243		2.4 ± 0.16	2.0 ± 1.1	0.93 ± 0.12	1.2 ± 0.23	2.0 ± 0.37	3.4 ± 1.3	ns
U7	unknown 7	1276		7.3 ± 1.5 ^a^	4.1 ± 2.1 ^ab^	1.0 ± 0.29 ^b^	0.66 ± 0.09 ^b^	2.2 ± 0.88 ^b^	3.2 ± 0.71 ^b^	**
U8	unknown 8	1450		12 ± 3.8 ^a^	3.3 ± 0.53 ^b^	nd ^b^	2.0 ± 0.34 ^b^	1.9 ± 0.48 ^b^	4.3 ± 0.50 ^b^	***
U9	unknown 9	1543		2.0 ± 1.7	0.38 ± 0.53	nd	0.22 ± 0.31	0.36 ± 0.50	nd	ns
U10	unknown 10	1652		5.5 ± 0.70 ^a^	1.3 ± 0.35 ^bc^	3.2 ± 0.62 ^b^	1.2 ± 0.86 ^c^	1.3 ± 0.31 ^bc^	1.7 ± 0.17 ^bc^	***
U11	unknown 11	1710		2.0 ± 0.50 ^a^	nd ^b^	nd ^b^	nd ^b^	nd ^b^	nd ^b^	***
U12	unknown 12	1758		2.1 ± 1.2 ^a^	0.27 ± 0.20 ^b^	0.18 ± 0.06 ^b^	0.19 ± 0.08 ^b^	0.87 ± 0.38 ^ab^	0.44 ± 0.31 ^ab^	*
U13	unknown 13	1842		1.4 ± 0.07 ^a^	0.69 ± 0.10 ^b^	0.11 ± 0.16 ^c^	nd ^c^	0.55 ± 0.10 ^b^	nd ^c^	***

^a^ Linear retention index on a DB-5 column. ^b^ A, mass spectrum and LRI agree with those of authentic compounds; B, mass spectrum (spectral quality value > 80 was used) and LRI agrees with reference spectrum in the NIST/EPA/NIH mass spectra database and LRI agree with those in the literature cited; $ tentatively identified, spectral quality value of 70 was used for this compound. ^c^ Estimated quantities (mg) collected in the headspace of celery samples containing 0.5 mL of saturated calcium chloride and filled up to 5 mL with HPLC-grade water, calculated by comparison with of 100 μg/mL propyl propanoate used as internal standard; internal standard was used to normalise chromatograms; means of three replicate samples are shown; means not labelled with the same letters are significantly different (*p* < 0.05) according to genotype and Tukey’s HSD multiple pairwise comparison; nd—not detected; ns—not significant probability obtained by ANOVA; * significant at the 5% level; ** significant at the 1% level; *** significant at 0.1% level.

**Figure 1:**
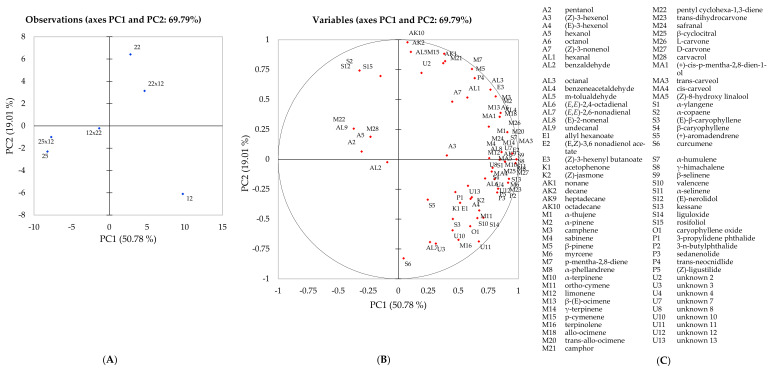
Principal component analysis of six celery samples showing correlations with volatile compounds: (**A**) projection of the samples; (**B**) distribution of variables; (**C**) compound codes as appear in plot (**B**).

Principal component analysis was used to visualise graphically the differences in the volatile composition of three parental genotypes and their hybrids and to examine any correlations occurring between genotypes (Figure 1). Using only the significant compounds according to the one-way ANOVA, a separation between genotypes was observed. Principal components one (PC1) and two (PC2) explained 69.79% of the total variation present within the data. Samples 12, 25, 25x12 and 12x22 were separated across F1, whereas samples 12, 22 and 22x12 along F2, respectively. The observation plot confirmed the findings presented in Table 2, where samples 12 and 22x12 expressed a strong association with many volatile compounds due to the high abundance identified. Conversely, samples 25 and 25x12, observed on the opposite side of the observation plot, displayed little or weak association with all volatile compounds (Figure 1). Due to the low abundance of volatile compounds, we can assume that these genotypes would be perceived as less aromatic when compared to the other genotypes. The hybrid 12x22 was positioned in the middle of the observation plot, displaying a stronger association with volatile compounds than genotype 25 and its hybrid 25x12; however, the relative abundance expressed within this hybrid remains consistently lower than 22x12 in all compound groups, except for phthalides. Thus, we could assume that this hybrid (12x22) was less aromatic than 22x12 but still had the typical, distinctive celery aroma. Comparing the aroma profile between the three parental genotypes and the hybrid lines, genotype 12 and hybrid 22x12 expressed the highest relative abundance of volatile compounds and it can be hypothesised that these will be more aromatic genotypes in comparison to the other samples. The current results (Table 1) confirmed previous work [12,13] where genotype 12 was shown to be very aromatic with strong flavour associations but low scoring in mouthfeel attributes such as crunchy and moist yet scored high for stringiness. Genotype 25 was reported to be less aromatic with a distinct cucumber flavour but was profiled as very crunchy, moist and with a firm first bite. The volatile content of genotype 22 was not significantly higher to genotype 12 or lower than 25 [12,13].

Overall, genotype 25 and hybrid 25x12 displayed clear maternal inheritance within the volatile content in terms of the compounds identified and their relative abundance. The high abundance of volatile compounds identified in genotype 12 appeared to have been inherited by hybrids 22x12 and 12x22 (Table 1). This relationship is also clear in the observation plot (Figure 1), where genotypes 12 and 22 with 22x12 and 12x22 expressing strong associations with all volatile compounds identified. We hypothesised that the parental genotypes would perform as previously [12,13] and maternal and paternal inheritance patterns become clearer upon sensory assessment, identifying phenotypic similarities between the parents and hybrids. Therefore, sensory evaluation was performed using a trained panel to further investigate these assumptions.

### 2.2. Sensory Evaluation of Celery Samples

The sensory profile of the three parental genotypes and hybrids was generated by a trained panel who came to the consensus of 28 terms for the quantitative assessment of celery samples and mean panel scores for these attributes are presented in Table 2. Out of the 28 attributes that were profiled, 15 of these were identified to be significantly different between genotypes. Few significant assessor x sample interactions were identified, suggesting that the panellists scored the samples in a consistent manner [41].

**Table 2:** Mean panel scores for sensory attributes of six celery samples.

Code	Attribute	Scores ^A^						*p*-Value ^B^
		12	25	22	25x12	22x12	12x22	
	**Appearance**							
CA	Colour	66.9 ^a^	31.1 ^d^	62.9 ^ab^	51.1 ^c^	59.6 ^abc^	55.6 ^bc^	***
STA	Stalk thickness (depth of cross-section)	25.2 ^c^	61.2 ^a^	60.0 ^a^	58.4 ^a^	45.4 ^b^	49.3 ^ab^	***
RA	Ribbed (well-defined ribs)	77.3 ^a^	52.5 ^d^	61.1 ^bc^	58.5 ^cd^	65.1 ^bc^	68.9 ^b^	***
	**Aroma**							
FFA	Fresh fennel	16.3	14.2	18	15.9	13.1	20	ns
GGA	Grassy/green	34.5 ^a^	19.9 ^b^	31.3 ^ab^	28.9 ^ab^	29.5 ^ab^	32.9 ^a^	**
FPA	Fresh parsley	23.7 ^a^	12.3 ^b^	22.3 ^ab^	13.1 ^ab^	23.4 ^ab^	16.8 ^ab^	**
FCA	Fresh coriander	14.5	10.5	16.9	16.7	13.2	14.2	ns
	**Taste/flavour**							
BT	Bitter	44.5 ^a^	26.0 ^c^	36.1 ^ab^	28.6 ^bc^	32.1 ^bc^	34.1 ^bc^	***
ST	Sweet	3.4 ^b^	11.7 ^a^	7.9 ^ab^	7.5 ^ab^	8.9 ^ab^	9.1 ^ab^	*
SAT	Salt	19.1	14.9	17.6	17.3	17.9	17.6	ns
UT	Umami	2.7	4	2.9	3.7	3.3	3.6	ns
FFF	Fresh fennel	15.8	12	20.3	15.7	15.7	23.5	ns
RF	Rocket	4.8	1.1	2.5	3.9	3.4	2.9	ns
FCF	Fresh coriander	16.1	14.5	18.9	18.7	13	16.8	ns
FPF	Fresh parsley	25.9 ^a^	9.8 ^b^	20.9 ^ab^	16.3 ^ab^	20.7 ^ab^	16.5 ^ab^	*
SF	Soapy	18.6	10.5	13.4	16.8	15.3	15.9	ns
GGF	Grassy/green	28.4	26.5	26.5	24.4	24.4	30	ns
	**Mouthfeel**							
CM	Crunchy	54.7 ^a^	55.4 ^a^	63.8 ^a^	65.7 ^a^	59.3 ^a^	63.2 ^a^	*
SM	Stringy	68.1 ^a^	45.2 ^b^	44.5 ^b^	55.3 ^ab^	54.4 ^b^	55.5 ^ab^	***
MM	Moist	42.6 ^c^	70.7 ^a^	67.5 ^a^	66.1 ^a^	53.6 ^b^	61.3 ^ab^	***
FM	Firmness of first bite	50.5 ^b^	54.5 ^ab^	62.3 ^ab^	62.2 ^ab^	54.4 ^ab^	65.2 ^a^	**
	**After-effects**							
CAE	Celery residue in the mouth	40.4 ^a^	29.9 ^b^	29.8 ^b^	31.9 ^b^	30.5 ^b^	34.5 ^ab^	***
NAE	Numbness	21.7 ^a^	10.3 ^b^	17.6 ^ab^	16.4 ^ab^	16.2 ^ab^	15.4 ^ab^	**
BAE	Bitter	31.9 ^a^	16.8 ^b^	23.9 ^ab^	22.9 ^b^	21.2 ^b^	22.3 ^b^	***
UAE	Umami	3.2	3.3	3.1	1.4	3.2	3.5	ns
SAE	Salty	13.5	11.7	11.8	12.9	12.6	13.4	ns
SOAE	Soapy	11.7	9.3	9.5	13.3	12.3	12.5	ns
GGAE	Grassy/green	27.1	21.2	21.9	20.8	21.5	24	ns

^A^ Means are from two replicate samples; differing small letters ^(a,b,c,d)^ represent sample significance from multiple comparisons and means not labelled with the same letters are significantly different (*p* < 0.05); nd, not detected. ^B^ Probability obtained by ANOVA that there is a difference between means; ns, no significant difference between means (*p* > 0.05); * significant at the 5% level; ** significant at the 1% level; *** significant at 0.1% level.

Appearance and mouthfeel attributes expressed the highest number of significant differences between genotypes. The appearance of the celery samples can be found in Table 9. Genotype 12 was scored high for appearance attributes (CA, RA) and hybrids descended from this genotype appear to have inherited these phenotypic characteristics, as high scores for both colour and ribbed were apparent. Their resemblance is also clear as shown in Table 9. Hybrid 22x12 displayed less prominent ribs and the scoring of this attribute was further decreased for 25x12 hybrid. Clearly, genotype 25 had a stronger influence on 25x12, where lower scores were observed for appearance. In terms of mouthfeel attributes, genotype 12 was shown to be the least crunchy, most stringy, with the driest petiole with a soft first bite. The genetic crosses appear to have these altered mouthfeel attributes, expressing higher scores for crunchiness, stringiness, and moistness. Hybrids 12x22 and 25x12 exhibited higher mean moistness and lower mean stringiness scores when compared to genotype 12. The data provide evidence of the influence of the female counterpart (the first number expressed in the hybrid cross) upon the appearance outcome of the offspring but when the male counterpart used displayed less prominent ribs (22 and 25), the ribbed appearance is reduced in the hybrids accordingly (Table 2).

Seven out of the ten odour and flavour attributes evaluated showed no significant differences between genotypes apart from grass odour and fresh parsley odour and flavour. Genotype 12 was scored significantly higher for grass and fresh parsley odour and flavour followed by genotype 22. The resemblance in scoring is reflected by the volatile content between these parents, whereby fewer significant differences were observed (Table 1). Although the genetic code of these genotypes was not revealed, it is possible that these parents are closely related as they share several characteristics. Investigating their hybrids, 12x22 displayed a high score for grass odour, like genotype 12, whereas 22x12 was scored high for fresh parsley odour and flavour as genotype 22. The parental genotype is closely associated with the descendent hybrid, with the hybrids expressing similar appearance, odour, and flavour characteristics (Table 2).

**Figure 2:**
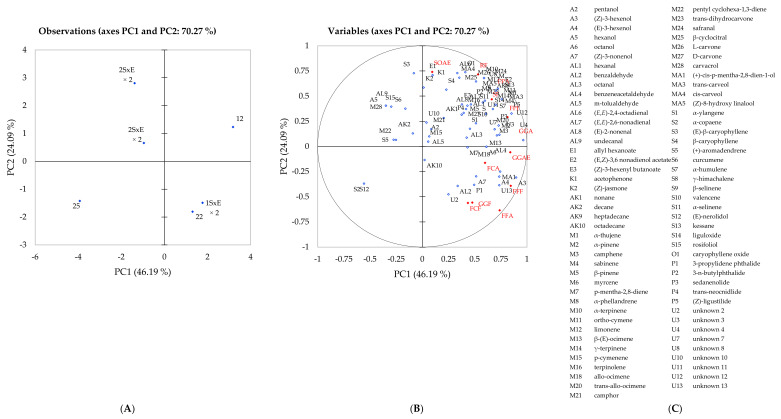
Principal component analysis of six celery samples showing correlations with volatile compounds and sensory profiling: (**A**) projection of the samples; (**B**) distribution of variables, sensory attributes are highlighted in red; (**C**) compound codes as appear in plot (**B**).

PCA was used to visualise the sensory and chemical differences observed across the genotypes and hybrids with the volatile compounds identified (Table 1) and odour and flavour attributes (Table 2) used as variables (Figure 2). Principal components one (PC1) and two (PC2) explained 70.27% of the total variation present within the dataset where the first axis separated genotypes 22, 25 and 12x22 and the second axis separated genotypes 12, 22 and 12x22, respectively. Genotypes 12 and 25 were displayed as opposites with genotype 12 expressing associations with many aroma compounds due to the high relative abundance identified and genotype 25 displayed no association with any flavour attribute due to its low relative abundance (Table 1). The profiling of genotypes 12 and 25 reflects previous studies, whereby both 12 and 25 were profiled as high and low extremes when grown in different geographical locations and across multiple years [12,13]. Throughout these experiments, these genotypes have represented the most significantly different genotypes for all sensory attributes as well as behaved consistently in terms of their volatile profile when grown in different geographical locations and across multiple years. For this reason, they were recommended as “stable” genotypes for fresh produce growers [9,12,13]. Genotypes 12, 22 and 12x22 were mostly associated with flavour and odour attributes including fresh fennel, coriander, and parsley and with most of the volatile compounds. Hybrid 25x12 expressed lower associations with these flavour attributes due to its lower relative abundance of monoterpenes, sesquiterpenes and phthalides and low scoring by the trained panel (Table 1 and Table 2).

The grass odour observed in the hybrid 12x22 was inherited from its female parent genotype 12, both expressing high relative abundance in (*Z*)- and (*E*)-3-hexenol, (*Z*)-3-hexenyl butanoate and (*E*,*Z*)-3,6-nonadienol acetate, compounds observed to express a fresh, grass-like odour. Whereas the fresh parsley odour observed in hybrid 22x12 was inherited from the female parent genotype 22, both expressing a high relative abundance of monoterpene compounds also identified in fresh parsley including α-pinene, camphene, *p*-mentha-2,8-diene and β-pinene [5,42] (Table 2). Along with this, genotype 12 was positively correlated with soapy flavour and the associations to flavour and odour attributes, combined with the high abundance of many volatile compounds (Table 1) confirms that genotype 12 is very aromatic. On the other hand, genotype 25 expresses no close association with any of the flavour and odour attributes confirming the previous statement that this genotype is not aromatic compared to genotype 12 or 22. Similar odour and flavour characteristics of genotype 25 were displayed in hybrid 25x12 (Figure 2, Table 2).

In terms of the sensory attributes, grass odour and flavour and parsley flavour were positively correlated with genotype 12, 22 and their hybrids. Alcohols (A3, A4), monoterpenes (M6, M11), sesquiterpenes (S13, S14) and phthalides (P3, P4) also displayed positive correlation with these samples and attributes. Fresh parsley odour and flavour that was scored highly in genotype 22 and hybrid 22x12 expressed a positive relationship with each other accompanied by; esters (E1, E2), monoterpenes (M1-M4, M6, M8, M10, M12, M14, M20, M23–27), sesquiterpenes (S7–S9, S11, S13) and phthalides (P2, P3) (Figure 2). Many compounds displayed a positive correlation with fresh parsley which was expected due to similarities between the celery and parsley aroma composition. Genotype 25 and hybrid 25x12 displayed the lowest scores of fresh parsley aroma and flavour due to the lower relative abundance of these compounds that were identified (Table 1).

The results presented in Table 1 and Table 2 showed significant differences in the aroma composition and sensory characteristics between the parental genotypes and hybrids and inherited characteristics were observed between parents and their offspring. Whether these celery hybrids meet the desires of the consumer, if there is a more preferred hybrid and what are the drivers of preference in celery was determined through the completion of a consumer trial, whereby the consumer acceptability of these hybrids and parental genotypes was investigated.

### 2.3. Consumer Evaluation of Celery Samples

One hundred and eighteen consumers evaluated the celery samples, and the demographic data are summarised in Table 3. A higher proportion of the consumers were female (63.6%), and the mean and median ages were 34.9 and 30, respectively. Close to half of the consumers were working (48.3%) and 47.5% were students. In total, 43.2% of consumers related to the food and nutrition department at the University of Reading. The largest ethnic group was White (English, Welsh, Scottish, Northern Irish or British), making up 42.4% of the sample population. Most consumers taking part stated that they liked celery (70.3%) and the most frequent consumption was less than once a month (45.8%).

The mean liking scores of the celery samples are presented in Table 4. The results demonstrated a significant difference in appearance, aroma, texture, and overall liking for all the samples that were tested, with results ranging from dislike slightly to like slightly. No significant difference was identified in taste liking for all samples and all samples were scored with an average score of 5; ‘neither like nor dislike’. While consumers did not like the celery samples extremely, the attributes of the hybrids, particularly 25x12 and 12x22, were scored higher for appearance, aroma and texture liking than the parental genotypes. Genotype 12 was scored the lowest for overall liking. When consumers were asked to rank the hybrids from the most liked (1) to least liked (3), no significant difference was observed; samples were scored at approximately 2, which demonstrated no significant preference.

Consumers were also asked to rank a list of six attributes that they found most important when consuming celery. The list that was presented to them contained attributes that are common in celery and in some cases, were very prominent in the samples such as the smooth exterior (not stringy). The attribute ‘crunchy’ was ranked as the most important followed by sweet taste, whereas the attribute bitter taste ranked as the least important when consuming celery (Table 5). Although ranked as least important, bitterness should still be considered an important characteristic to celery taste as the compounds that inflict bitterness and astringency often possess multiple health benefits upon consumption including antioxidant, anti-inflammatory, and anticancer properties [43,44,45]. These are predominately from non-volatile compounds such as phenolic acids and flavonoids [43,44,45].

#### Agglomerative Hierarchical Cluster Analysis of Consumer Data and Internal Preference Mapping

Agglomerative hierarchical cluster (AHC) analysis was completed to identify relatively homogeneous groups of consumers based on their overall liking scores. Three clusters of consumers were identified and the mean liking scores of the clusters are presented in Table 6. Consumers in cluster 1 (43.2%) neither liked or disliked hybrids 25x12 and 22x12 and expressed a moderate dislike for genotype 12. Cluster 2 (38.9%) behaved in a similar manner to cluster 1, liking slightly genotypes 25, 22 and 25x12 and neither liked or disliked genotype 12 and hybrid 22x12. Opposing clusters 1 and 2, consumers in cluster 3 (17.8%) liked slightly genotype 12 and moderately disliked 25x12 due to its strong flavour attributes. 

Labelling each participant present within each cluster as a liker or non-liker, 60.8, 82.6 and 57.1% were celery likers in clusters 1, 2 and 3. Interestingly, cluster 3 contained the highest proportion of celery non-likers and they liked the most genotype 12, a genotype that expressed a high abundance of volatile compounds and profiled as very aromatic with a strong bitter taste, whereas 25x12 was the least liked and profiled as less aromatic (Table 2). On the other hand, hybrid 25x12 was the most liked of the hybrids according to clusters 1 and 2. One reason might be the high score of crunchiness and moist mouthfeel by the trained panel (Table 2); both attributes ranked as important according to consumers (Table 5). There was also significant interaction between sample x cluster for overall liking confirming that consumers scored differently the samples in each cluster (Table 6). 

**Figure 3:**
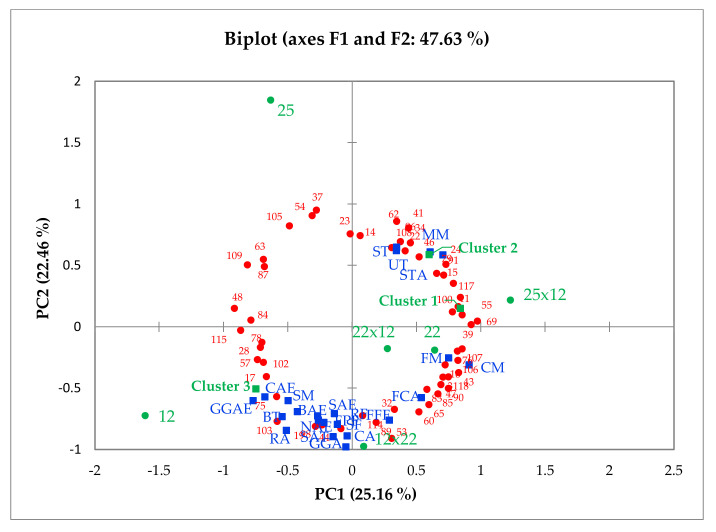
Internal preference map of six celery samples. Sensory attributes and consumer cluster means were regressed onto the consumer preference matrix generated by PCA. Blue squares—sensory attributes, codes correspond to those in Table 2. Green squares—clusters 1, 2, 3, mean liking positions of three clusters from AHC (Table 6). Red circles: overall liking scores of each consumer.

Sensory attributes assessed by the trained panel (Table 2) and mean liking scores of each cluster were regressed onto the first two principal components of the consumer overall liking data to form an internal preference map (Figure 3). Principal components one (PC1) and two (PC2) explained 47.63% of the variation in the data with hybrids and genotype 22 separated from genotypes 12 and 25 across PC1, driven by sweet taste (ST), moist mouthfeel (MM) and stalk thickness (STA) attributes. Genotypes 12 and 25 were separated across PC2 with genotype 12 being positively correlated with grass/green flavour (GGF), bitter taste (BT) and stringy mouthfeel (SM) attributes.

Cluster 1 displayed no significant relationship with any sensory characteristics (Figure 3), therefore, confirming that celery not possessing a strong aroma such as hybrids 22x12 and 25x12 (Table 1 and Table 2), were more liked. Genotypes 25 and 22 and hybrid 25x12 were scored highly for stalk thickness (STA), moist mouthfeel (MM) and had a firm first bite (FM) with a sweet taste (ST) as discussed during sensory profiling (Table 2) and these attributes were closely associated to the most liked genotypes within cluster 2. Both clusters expressed no significant correlation with any flavour or odour attributes and preferred the celery that expressed low relative abundance of the volatile compounds (Table 1). For this reason, genotype 12 was the most disliked celery sample for clusters 1 and 2. Genotype 12 expressed a high relative abundance of volatile compounds (Table 1) in addition to scoring significantly higher in grass/green flavour (Table 2). Ribbed appearance (RA), grass/green aroma (GGA), bitter taste (BT) and fresh parsley aroma and flavour (FPA and FPF) were attributes positively correlated with this genotype.

Clusters 1 and 2 displayed similar overall liking scores in comparison to cluster 3. However, observed in the bottom right quadrant there appears to be a ‘gap’ where none of the clusters are placed (Figure 3) yet genotype 22 and hybrids 22x12 and 12x22 are positioned there. Although no cluster were associated with these hybrids, the consumers that are situated there displayed preference to celery that expressed a fresh fennel flavour and aroma accompanied by a soapy aftertaste. Hybrid 25x12 was the closest match to the highest proportion of consumers that were grouped into clusters 1 and 2. However, the hybrid requires further development with particular focus on the moist mouthfeel, stalk thickness and sweet taste attributes. These attributes are the drivers of liking for 82% of the consumers in this study. On the other hand, the drivers of liking for those consumers placed in cluster 3 (18%) were grassy flavour and bitter taste.

Penalty analysis was used to relate Just-About-Right (JAR) data to liking scores and explain drivers of overall liking in relation to aroma, sweetness, bitterness, flavour and stringiness intensity and the results are presented in Table 7. 

When the attributes are not at the optimum intensity for a consumer this may influence the overall liking. Sweetness was ranked by the consumers as the second most important characteristic, and this was reflected in Table 7, whereby for all genotypes and hybrids, there was a negative impact on the overall liking when the sweetness of the samples was considered too low. This agreed with over 50% of the consumers in all samples. On the other hand, there was a significant drop in the liking of all samples when the bitter taste intensity was “too much” by the consumers with the genotypes 12 and 22 perceived the most bitter and genotype 25 the least bitter. Hybrid samples were scored in between the parent genotypes. Interestingly, regarding the flavour intensity attribute, it can be observed that there was a significant drop in the liking for almost all samples when the flavour intensity of the samples was considered either “too little” or “too much”. Where significant drops were observed for flavour intensity attribute, no significant drop in overall liking was observed for aroma intensity, too little or too much, displaying that consuming celery is more important for deciding preference than just smelling the sample. Stringiness, which expressed a negative correlation with crunchy texture by the sensory panel (Table 2), displayed significant drops in overall liking if samples were considered to be “too much” in genotype 12 and all the hybrids. Genotype 12 and hybrid 12x22 were considered to be the most stringy, and a mean drop of 1.3 and 0.9 in the overall liking occurred, respectively. Although scored lower, the stringiness scored by the panel of 12x22 was like genotype 12 (Table 2). The maternal inheritance of the ribbed appearance is clearly demonstrated from genotype 12 in 12x22. As texture was scored as an important attribute for consumers (Table 5), we would recommend to breeders to use a female parent that expresses the desirable appearance and textural attributes as a strong maternal inheritance has been observed in this study.

Additional comments on the samples provided by the participants contained both positive and negative points and these are shown in Table 8. Although bitter and sweet taste have been identified as drivers of disliking and liking, the results from the consumer evaluation of celery samples demonstrated that consumers could not identify differences in taste (Table 4) whereas the trained panel clearly identified significant differences between all samples in sweetness and bitterness (Table 2).

Overall, there was no hybrid that was significantly preferred by the consumer with all hybrids scoring between 2.0 and 2.1 (Table 4). Both 25x12 and 22x12 were scored in a similar manner in preference ranking (Table 4) as well as in sensory analysis; however, upon combining the data collected from liking (Table 4), attribute ranking (Table 5), cluster analysis (Table 6) and JAR (Table 7), with further developing, 25x12 holds the potential to be a new hybrid that matches most of the consumers’ desire. Expressing characteristics including a crunchy and moist mouthfeel, low stringiness and an odour and flavour that was not scored too highly by the panel (Table 1 and Table 2, Figure 1 and Figure 2). Contrastingly, hybrid 12x22 expressed high abundance of volatile compounds (Table 1) and was scored accordingly by the panel, with strong associations to fresh parsley flavour (Figure 2 and Figure 3). The maternal inheritance was clear in both 12x22 and 25x12, with the characteristics of both female parents displayed within the hybrids. This was less apparent in hybrid 22x12, whereby the possibility of these genotypes being closely related causes difficulties with matching parental characteristics. The overall liking score for genotype 12 was the lowest (Table 4), possibly due to the sample expressing a stringy and dry mouthfeel attributes yet high scoring flavour attributes such as soapy, fresh parsley and grass (Table 2). This genotype was also scored as the most bitter and least sweet. Bitterness was an attribute ranked as least important and sweetness was ranked as second most important for consumers, when considering their most desirable characteristics for a celery (Table 5). 25x12 was the only hybrid that expressed a mean drop in liking if an increase or decrease in bitterness occurred (Table 7) possibly indicating that the bitter intensity of this crop is at an acceptable level for 21% of consumers. This hybrid contains genetic material from both genotypes 25 and 12, the most sweet and bitter parental genotypes, and we can clearly see that the favourable attributes of both genotypes have been passed on; the preferred mouthfeel attributes of genotype 25 combined with the distinct flavour of genotype 12 without being overpowering. The taste characteristics have been combined to produce a less bitter hybrid.

## 3. Materials and Methods

### 3.1. Celery Material and MIAPAE Standard

#### 3.1.1. Sample Information

The three parental genotypes used in this experiment were chosen due to their differences in physical and chemical attributes and the original genetic crosses of the hybrid were carried out in 2018 at Tozer Seeds Ltd. (Pyports, UK). Although commercial confidentiality precludes revealing the exact genetic identity of each genotype used in this paper, the origins of the parental breeding lines and their image postharvest are presented in Table 9. 

#### 3.1.2. Timing, Location and Environment

Celery seed (*Apium graveolens*) of eight parental genotypes supplied by Tozer Seeds Ltd. (Cobham, UK) were grown in commercial conditions and harvested in El Albujon, Murcia, Spain 2021 (37°43′05.5″ N 1°03′24.3″ W). Plugs were transplanted after 56 days growing in a nursery and then harvested 113 days later. Plants were lifted, packed, and despatched on the same day. Average daily air temperature was 17.7 °C, with 1.0 mm average daily rainfall; average relative humidity was 81.5%, with an average daily wind speed of 6.3 m/s.

#### 3.1.3. Raw Material Collection, Processing Storage

The celery was grown in three randomised blocks in the centre of the field to reduce any influence from edge effects at a density of 10 plants per m^2^ and three replicates were harvested from each block using a celery knife. Celery petioles were cut to 20 cm, discarding outer petioles, the base, leaves and any knuckles and sealed in labelled freezer bags with freezer blocks for transportation to the UK. Samples arrived in the UK within two days postharvest. Celery samples used for sensory and consumer evaluation were refrigerated for two further days. Samples for aroma analysis were refrigerated for two days before analysis. Panel and consumer tasting occurred on the same day as aroma analysis (P + 4). 

### 3.2. Chemical Reagents

For GC–MS analysis, calcium chloride and the alkane standard C_6_–C_25_ (100 μg/mL) in diethyl ether were obtained from Merck (Poole, UK).

### 3.3. Volatile Analysis Using SPME GC–MS

Prior to analysis, the fresh celery sample was macerated, and a 2 g sample was combined with 0.5 mL of saturated calcium chloride solution and filled up to 5 mL with HPLC-grade water in a 15 mL SPME vial fitted with a screw cap lid. After equilibration at 37 °C for 10 min, a 75 µm DVB/CAR/PDMS fibre (Supelco, Bellefonte, PA, USA) was exposed to the headspace above the samples for 30 min. Throughout equilibration and fibre exposure, the sample was constantly agitated at a rate of 500 rpm. Samples were analysed by automated headspace SPME using an Agilent 110 PAL injection system and Agilent 7890 gas chromatograph with 5975C mass spectrometer (Agilent, Santa Clara, CA, USA) with a DB5 column (30 m × 0.25 mm × 0.25 µm) from Agilent (Palo Alto, CA, USA) and the identification of volatile compounds was conducted as described by Turner et al. [9].

### 3.4. Sensory Profiling

Sensory evaluation was carried out using quantitative descriptive analysis (QDA^TM^) to determine the sensory characteristics of the celery samples and the characteristics were estimated quantitatively as suggested by Stone, Sidel, Oliver, Woolsey and Singleton [46]. The trained sensory panel at the Sensory Science Centre (University of Reading, *n* = 12; 11 female and 1 male) was used to develop a consensus vocabulary to describe the sensory characteristics of the three celery genotypes and three celery hybrids. During the development of the sensory profile, the panellists were asked to describe the appearance, odour, taste, flavour, mouthfeel and aftereffects of the samples in order to produce as many descriptive terms as seemed appropriate. References were used to help confirm the characteristics of certain attributes including fresh and dried fennel, salad rocket, flat leaf parsley and fresh coriander. The terms were discussed by the panellists as a group, with the help of the panel leader, and this led to a consensus of 28 attributes. Due to the COVID-19 pandemic restrictions, the trained panel assessed the samples from home. Vocabulary refreshment and training sessions occurred prior to scoring virtually on the Teams platform. Samples were prepared and were sent out to panellists using chilled transport couriers. The panellists completed their scoring simultaneously using Compusense Cloud software (Version 21.0.7713.26683, Compusense, Guelph, ON, Canada) whilst on video on Teams. Celery petioles presented to the panellists were chosen to be as uniform as possible. The first outer petioles were removed and discarded. The next ring of petioles was used, and these were washed with filtered water and cut to 15 cm petiole length. The panellists scored in duplicate for each sample in separate sessions. Samples, coded with three-digit random numbers, were provided in a monadic balanced order, with sample sets randomly allocated to panellists. The panellists were asked to assess the appearance first; to break the petiole in half to assess the odour; to bite from the middle for taste, flavour and mouthfeel; and then after 30 s delay to assess the aftereffects. The intensity of each attribute for each sample was recorded on a 100-point unstructured line scale. Between samples, the panellists cleansed their palate with water and crackers.

### 3.5. Consumer Evaluation

One hundred and eighteen volunteers were recruited across the University of Reading (male and female, aged 18 years and above, non-smokers and without allergies or intolerances to wheat, gluten and/or celery). This study was performed as an at-home study due to ongoing COVID-19 restrictions, complying with social distancing and COVID-19 guidelines, as well as risk assessments in place. This study was fully explained to the volunteers and their informed written consent was obtained prior to participation. Participants collected their samples from the Sensory Science Centre (University of Reading) along with palate cleanser (crackers) and other information regarding how to access this study online. Participants were asked to complete this study within 24 h and keep the samples refrigerated until ready to begin the test. Participants were asked, after observing the samples, to rate their liking (appearance, aroma, taste, texture and overall) on a 9-point hedonic scale (where 1: dislike extremely, 5: neither like nor dislike, 9: like extremely) for all samples. They also indicated the appropriateness of attribute level on a 5-point Just-About-Right (JAR) scale for the following attributes: aroma intensity, bitterness, sweetness, flavour intensity and stringiness (where 1: much too low, 3: JAR and 5: much too strong). Participants were asked to indicate their preference for the hybrid genotypes only (25x12, 22x12 and 12x22) and rank various celery characteristics such as smooth exterior, moist texture, crunchy texture, sweet taste, bitter taste, and strong aroma (from most important to least important). Finally, participants were asked a series of demographic questions, purchase intent and celery consumption and were given the opportunity to leave additional comments after evaluating each sample if they wanted to. In total, six samples were evaluated (three parental genotypes and three celery hybrids in one session). Samples were presented to participants in a monadic balanced order using William’s design, with sample sets randomly assigned to consumers. Data were collected using Compusense Cloud Software (Version 21.0.7713.26683, Compusense, Guelph, ON, Canada). The School of Chemistry, Food and Pharmacy Research Ethics Committee (SREC) provided a favourable opinion for conduct (SREC 11/2021) and this study was conducted in March 2021.

### 3.6. Statistical Analysis

Quantitative data for all compounds identified in the SPME GC–MS analysis were analysed by one-way analysis of variance (ANOVA) and principal component analysis (PCA) using XLSTAT Version 2020.1.3 (Addinsoft, Paris, France). For those compounds exhibiting significant difference in the one-way ANOVA, Tukey’s Honest Significant Difference post hoc test was applied to determine which sample means differed significantly (*p* < 0.05) between the celery genotypes. Only those compounds exhibiting significant differences between genotype were included in the principal component analysis. 

SENPAQ version 6.3 (Qi Statistics, Kent, UK) was used to carry out ANOVA of sensory panel data, where the main effects (sample and assessor) were tested against the sample by assessor interaction with sample as a fixed effect and assessor as a random effect. The means from sensory data were taken over assessors and correlated with the relative abundance means from the instrumental data via PCA using XLSTAT (Version 2020.1.3 (Addinsoft, Paris, France)). Internal preference mapping was used to relate sensory characteristics of celery samples to consumer liking data. XLSTAT was used to carry out the following analyses: (i) PCA of the volatile and sensory panel data, (ii) one-way ANOVA for the aroma analysis and consumer liking, (iii) analysis of the preference (ranking) data using Friedman’s test, (iv) agglomerative hierarchical clustering (AHC) for overall liking, (v) penalty analysis of the JAR data and (vi) internal preference mapping. In more detail, for the AHC, dissimilarity of responses was determined by Euclidean distance, and agglomeration using Ward’s method (set to automatic truncation). Sample by cluster interactions were also tested by two-way ANOVA. For the penalty analysis, the influence of consumer perception of appropriateness of attribute level rating (JAR) on consumer liking was evaluated by calculating the mean drop in liking rating (scale 1–9) compared with mean liking of consumers that rated the attribute as JAR (JAR 3 on a 1–5 scale), determining whether this drop in liking score was significant.

## 4. Conclusions

The present study aimed to explore the sensory characteristics of new celery hybrids and their parental genotypes, identifying similarities and differences between the parents and offspring, and to evaluate consumer liking and perceptions of celery hybrids. Significant differences between parental genotypes and hybrids were observed in the aroma composition, sensory profiling, and consumer liking. In addition, non-significant differences were observed in parent genotypes and their hybrid off-spring highlighting the potential for maternal and paternal inheritance of phenotypic characteristics. 

The hybrids in this study were grown in Spain (2021) and before we can confirm with confidence that we have developed a celery variety that meets the consumer demands, these hybrids must be grown in different scenarios and investigate any variation occurring within the aroma composition and changes in the sensory characteristics. Growing these hybrids in different geographical locations and over multiple years will identify the stability of these hybrid lines and examine how variables including air temperature, soil type, water composition and different agronomical techniques might influence the aroma profile. Following this up with sensory profiling will identify the impact of these variables upon the aroma composition and consumer preference for the hybrids. 

The findings from this study combined with previous studies completed by the authors will contribute to further understanding how changes in the aroma and sensory profile may influence consumer acceptability and preference. This work provides knowledge and pinpoints the importance of attributes that drive consumer preference which in turn is useful to fresh produce growers and breeders. Furthermore, the information on the maternal inheritance of characteristics in celery has been displayed in this paper will aid breeders in the understanding of inheritance in celery, ultimately leading to the production of new celery hybrid lines that are consumer preference-driven based on their metabolite and sensory profile.

## Figures and Tables

**Table 3:** Consumer demographics and characteristics of the consumer panel.

Consumers	Number	Percentage (%)
Total number of volunteers	118	
Age		
mean	34.9	
median	30	
min	19	
max	71	
Gender		
male	42	35.6
female	75	63.6
prefer not to say	1	0.84
Working Status		
working	57	48.3
unemployed	3	2.5
student	56	47.5
other	2	1.7
working in food/nutrition/sensory sector	51	43.2
Ethnic group		
White	73	61.9
Mixed or Multiple ethnic groups	2	1.7
Asian or Asian British	21	17.8
Black, African, Caribbean or Black British	15	12.7
other ethnic group	7	5.9
Celery liking		
yes	83	70.3
no	35	29.7
Consumption Frequency		
less than once a month	54	45.8
once a month	19	16.1
2 to 3 times per month	19	16.1
once a week	13	11
2 to 4 times per week	9	7.6
once a day	4	3.4
Purchase Frequency		
once a month	80	67.8
once a week	17	14.4
never	21	17.8
Method of consumption		
I do not eat celery	15	12.7
raw (on its own)	25	21.2
raw (with condiments)	49	41.5
raw (in salads)	42	35.6
cooked (boiled, roasted, fried, on its own)	47	39.8
cooked (in soups, stocks or sauces)	68	57.6
other	6	5.1

**Table 4:** Liking scores and preference ranking for celery samples.

Samples	Liking ^A^					Ranking ^B^
	Appearance	Aroma	Taste	Texture	Overall	
12	5.7 ^bc^	6.2 ^a^	5.0	4.7 ^c^	4.7 ^b^	-
25	5.0 ^c^	5.5 ^b^	5.3	6.0 ^ab^	5.5 ^a^	-
22	6.3 ^ab^	6.1 ^a^	5.3	6.6 ^a^	5.5 ^a^	-
25x12	6.1 ^b^	6.1 ^ab^	5.4	6.1 ^ab^	5.6 ^a^	2.0
22x12	6.3 ^ab^	6.1 ^ab^	5.4	5.8 ^b^	5.4 ^ab^	2.0
12x22	6.8 ^a^	6.2 ^ab^	5.4	6.1 ^ab^	5.6 ^a^	2.1
*p*-value ^C^	***	*	ns	***	**	ns

^A^ Means not labelled with the same letters ^(a,b,c)^ are significantly different (*p* < 0.05); means are from 118 consumers on a 9-point hedonic scale (from dislike extremely to like extremely). ^B^ Mean rank (1: most preferred to 3: least preferred). ^C^ ns, no significant difference between means (*p* > 0.05). ;* significant at the 5% level; ** significant at the 1% level; *** significant at 0.1% level.

**Table 5:** Consumers’ ranking for important attributes when consuming celery.

Attributes	Ranking ^A^
Crunchy texture	2.3 ^a^
Sweet taste	2.8 ^ab^
Moist texture	3.8 ^c^
Smooth exterior (not stringy)	3.4 ^bc^
Strong aroma	4.1 ^d^
Bitter taste	4.6 ^cd^

^A^ Mean rank (1: most important to 6: least important). Means not labelled with the same letters ^(a,b,c,d)^ are significantly different (*p* < 0.05).

**Table 6:** Overall liking of the celery samples for the cluster of consumers obtained from agglomerative hierarchical clustering.

Cluster/Percentage of Consumers	Samples ^1^						*p* Value ^2^	Overall Liking per Cluster ^3^
	12	25	22	25x12	22x12	12x22		
1 (43.2%)	3.5 ^c,AB^	4.6 ^ab,ABCD^	4.5 ^b,ABC^	5.5 ^a,CDEFGH^	5.2 ^ab,CDEF^	5.0 ^ab,CDE^	***	4.7 ^c^
2 (38.9%)	5.4 ^b,CDEFG^	6.8 ^a,H^	6.8 ^a,H^	6.7 ^a,GH^	5.7 ^b,CDEFGH^	6.1 ^ab,EFGH^	***	6.2 ^a^
3 (17.8%)	6.5 ^a,FGH^	4.8 ^bc,BCDE^	5.2 ^ab,CDEF^	3.3 ^c,A^	5.1 ^ab,CDEF^	6.0 ^ab,DEFGH^	***	5.1 ^b^
Overall liking per sample ^4^	4.7 ^b^	5.5 ^a^	5.5 ^a^	5.6 ^a^	5.4 ^ab^	5.6 ^a^		

^1^ Significant differences for the means per cluster (*p* < 0.05) within a row are denoted by differing small letters ^(a,b,c)^; means are from 51 consumers for cluster 1, 46 consumers for cluster 2 and 21 consumers for cluster 3, respectively; significant differences from the interaction (sample x cluster) are denoted by differing capital letters ^(A,B,C,D,E,F,G,H)^. ^2^ ***, significant at 0.1% level. ^3^ Mean for overall liking per each cluster was significantly different with *p* < 0.0001. ^4^ The mean for overall liking per sample is from 118 consumers and it was significantly different with *p* = 0.0004. Significant interaction between sample x cluster was observed as calculated by two-way ANOVA (*p* < 0.0001).

**Table 7:** Mean Just-About-Right ratings and penalty analysis showing the influence on overall liking ratings.

Samples	Overall ^A^	Significance of Sample (*p*-Value) ^B^	Penalty Analysis			
			Too Little		Too Much	
			Mean Drop	Frequency (%)	Mean Drop	Frequency (%)
	JAR Aroma					
12	2.9 ^a^	**	0.69	24.6	1.15	17.0
25	2.5 ^b^		0.49	48.3	3.30	7.6
22	2.8 ^a^		0.70	29.7	1.54	11.9
25x12	2.7 ^ab^		0.39	31.1	1.32	13.6
22x12	2.8 ^a^		0.61	30.5	1.62	13.6
12x22	2.9 ^a^		0.74	28.0	1.55	15.3
JAR Bitterness						
12	3.4 ^a^	**	1.15	15.3	2.09 *	45.8
25	2.9 ^b^		0.72	28.0	2.17 *	22.9
22	3.3 ^a^		1.45	14.4	2.09 *	40.7
25x12	3.1 ^ab^		0.60 *	21.2	1.98 *	30.5
22x12	3.2 ^ab^		0.52	21.2	1.56 *	33.9
12x22	3.2 ^ab^		0.51	21.2	2.22 *	30.5
JAR Sweetness						
12	2.2	ns	1.18 *	66.1	0.53	1.7
25	2.5		1.545 *	50.9	0.06	4.2
22	2.4		1.31 *	52.5	-	0.0
25x12	2.4		1.69 *	50.9	0.41	2.0
22x12	2.4		1.73 *	54.2	2.36	0.9
12x22	2.4		1.76 *	46.6	1.44	0.9
JAR Flavour						
12	3.3 ^a^	***	1.11	17.8	2.26 *	41.5
25	2.8 ^b^		1.37 *	38.1	2.75	15.3
22	3.0 ^ab^		1.26 *	23.7	2.28 *	40.7
25x12	3.1 ^ab^		1.10 *	24.6	2.39 *	28.8
22x12	3.0 ^ab^		1.16 *	22.9	1.96 *	25.4
12x22	3.1 ^ab^		1.26 *	22.0	2.39 *	30.5
JAR Stringiness						
12	4.0 ^a^	***	1.76	5.1	1.33 *	70.3
25	3.2 ^cd^		0.71	19.5	0.60	30.5
22	3.0 ^d^		−0.57	22.9	0.59	22.0
25x12	3.4 ^bc^		0.24	15.3	0.88 *	42.4
22x12	3.5 ^b^		−0.19	14.4	0.90 *	49.2
12x22	3.3 ^bcd^		0.62	11.9	1.64 *	35.6

^A^ Means not labelled with the same letters ^(a,b,c,d)^ are significantly different (*p* < 0.05). ^B^ Represents a significant difference (*p* < 0.05) within a sample in overall liking compared with mean liking rating when the sample was considered Just-About-Right; * significant at the 5% level; ** significant at the 1% level; *** significant at 0.1% level.

**Table 8:** Examples of participants’ comments (three positive and three negative comments) relating to the celery samples used in this study.

Sample	Comments and Participants Details
12	Very different from any other celery I had before. This is very yummy (IP12). Flavours were balanced and texture and appearance were good and appealing (IP120). It is very good fresh smell (IP63). Would not be pleased if I had bought this Did not finish it (IP3). I was unable to break it in two due to the fibres. It was excessively stringy, and the flavour was too strong too (IP32). It was very stringy. The aroma and taste was herbal (IP62)
25	Had a slight salty taste which I liked (IP117). This one is very juicy (IP65). Good texture and light overall flavour (IP19). Looked very pale. Bland flavour (IP51). Too pale in colour (IP112). I would not buy this because of the colour (IP88).
22	Very juicy in texture (IP14). This sample will be a good quality celery that I’m expecting when buying one (IP31). what I would expect from a good celery stick (IP49). No distinct flavour (IP59). Unpleasant after taste (IP110). Really bitter and salty (IP77)
25x12	Beautiful sample of celery (IP52). Overall good celery to taste and flavour (IP30). Crunchy and juicy (IP96). Very sweet and aromatic. Too stringy (IP116). Too stringy and rather boring overall (IP28). Too bitter, unpleasant (IP98).
22x12	Attractive celery, good cross section, and colour. Good crunch and mouthfeel not as stringy as many (IP09). I enjoyed this one was quite good and not as stringy as some of the other flavour was good and have a nice crunch (IP70). It looks more appealing (IP21). Flavour too strong and too stringy (IP7). This sample is stringy for me. Some fibres are left in mouth (IP40). This one is too stringy and bitter (IP75).
12x22	Very strong aroma and flavour. Texture and lack of strings was good. Nice colour (IP11). Really liked this sample, tastes of what celery to me should taste like (IP28). Good texture and flavour. My favourite (IP122). The intense taste bothered me. It tasted bitter at the first bite (IP83). Tasted very chemical-like (IP44). Very bitter aftertaste (IP36).

**Table 9:** Images of the petioles of the six celery samples used in this study.

	Samples					
Line	12	22	25	12x22	22x12	25x12
Origin	UK	USA	EU	-	-	-
Appearance	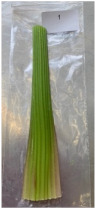	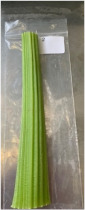	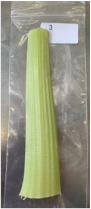	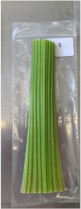	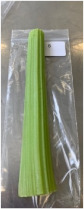	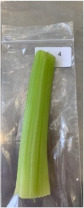

## Data Availability

The data presented in this study are available upon request from the corresponding author.
